# High-Quality Nucleic Acid Isolation from Hard-to-Lyse Bacterial Strains Using PMAP-36, a Broad-Spectrum Antimicrobial Peptide

**DOI:** 10.3390/ijms22084149

**Published:** 2021-04-16

**Authors:** Hye-sun Cho, Munjeong Choi, Yunjung Lee, Hyoim Jeon, Byeongyong Ahn, Nagasundarapandian Soundrarajan, Kwonho Hong, Jin-Hoi Kim, Chankyu Park

**Affiliations:** Department of Stem Cell and Regenerative Biotechnology, Konkuk University, Hwayang-dong, Gwangjin-gu, Seoul 05029, Korea; chssky77@gmail.com (H.-s.C.); alooomjc@gmail.com (M.C.); yunjungee98@gmail.com (Y.L.); kamuijhi@naver.com (H.J.); ahn.b@outlook.com (B.A.); sundarmeets@gmail.com (N.S.); hongk@konkuk.ac.kr (K.H.); jhkim541@konkuk.ac.kr (J.-H.K.)

**Keywords:** antimicrobial peptides, PMAP-36, nucleic acids isolation

## Abstract

The efficiency of existing cell lysis methods to isolate nucleic acids from diverse bacteria varies depending on cell wall structures. This study tested a novel idea of using broad-spectrum antimicrobial peptides to improve the lytic efficiency of hard-to-lyse bacteria and characterized their differences. The lysis conditions of *Staphylococcus aureus* using recombinant porcine myeloid antimicrobial peptide 36 (PMAP-36), a broad-spectrum pig cathelicidin, was optimized, and RNA isolation was performed with cultured pellets of ten bacterial species using various membranolytic proteins. Additionally, three other antimicrobial peptides, protegrin-1 (PG-1), melittin, and nisin, were evaluated for their suitability as the membranolytic agents of bacteria. However, PMAP-36 use resulted in the most successful outcomes in RNA isolation from diverse bacterial species. The amount of total RNA obtained using PMAP-36 increased by ~2-fold compared to lysozyme in *Salmonella typhimurium*. *Streptococci* species were refractory to all lytic proteins tested, although the RNA yield from PMAP-36 treatment was slightly higher than that from other methods. PMAP-36 use produced high-quality RNA, and reverse transcription PCR showed the efficient amplification of the 16S rRNA gene from all tested strains. Additionally, the results of genomic DNA isolation were similar to those of RNA isolation. Thus, our findings present an additional option for high quality and unbiased nucleic acid isolation from microbiomes or challenging bacterial strains.

## 1. Introduction

Rapid progress in high-throughput omics technologies has led to genome and transcriptome analyses, microbiome assays, and DNA-based rapid diagnoses of infectious diseases becoming essential tools for microbiological studies. However, the successful outcomes of these analyses often depend on the reliable extraction of high-quality nucleic acids from diverse microorganisms showing significant differences in their cell wall structures.

Bacterial cells are surrounded by cell surface structures that allow them to thrive in extreme environments. Some of the most important cell wall components are polysaccharide structures, including peptidoglycan (PGN), lipopolysaccharide (LPS), and extracellular polysaccharides [1]. Studies have reported significant diversity in bacterial cell wall structures and consequently their resistance to cell lytic agents [2] and antibiotics [3]. The preparation of genomic DNA or total RNA from hard-to-lyse bacteria could be extremely less efficient compared to easy-to-lyse bacteria. Therefore, various lysis protocols for bacteria, including chemical [4], enzymatic [5] and mechanical methods [6] or a combination of these [7], have been described.

Bacteriolytic enzymes, such as lysozyme, mutanolysin, and lysostaphin, have been used to disrupt cells. The major applications of these enzymes are related to the extraction of nucleic acids from susceptible bacteria and spheroplasting for cell transformation [5,6,7]. For example, Gram-positive bacterial species are often refractory to lysozyme [2], and lysostaphin cleaves the cross-linking pentaglycine bridges mainly in the cell wall of staphylococcal strains [8,9]. A detailed understanding of bacterial cell wall structures has been limited to only a few species [10]. Therefore, no lytic enzyme has shown sufficient activity against *Bacillus* spores and *Mycobacterium tuberculosis* [11,12].

Chemical or enzymatic breakdown of cell walls or membranes is often less efficient than expected in hard-to-lyse cells, although combined enzymatic and chemical lysis approaches can improve the results [13,14]. Mechanical lysis methods, such as bead beating, could be effective for hardy microbes, but bead beating has certain limitations, including the need for specialized equipment, variable extraction efficiencies, heating of samples, degradation of cellular products, and biohazard risks that arise from the creation of infectious aerosols [4,15].

Antimicrobial peptides (AMPs) are molecules produced by virtually all organisms as part of the innate immune system in multicellular organisms and involved in self-protection and microbial competition in microorganisms [16,17,18]. They exhibit strong antimicrobial activity against a broad range of microorganisms, including conventional antibiotic-resistant strains, with a rare possibility of inducing bacterial resistance [19]. Bacteriocins are bactericidal peptides produced by bacteria, as a self-protection mechanism that helps the microorganisms to survive in their natural habitats [16]. The main mechanism underlying the function of these peptides is the disruption of bacterial membranes [20,21]. Therefore, to the best of our knowledge, this is the first study to exploit the possible use of AMPs to break and open hard-to-lyse bacteria and test their applicability as a new membranolytic agent for the isolation of bacterial nucleic acids.

Porcine myeloid antimicrobial peptide 36 (PMAP-36; PMAP36) and melittin found in honeybee venom have been known as highly potent and broad-spectrum AMPs with a positively charged alpha helical structure [22,23]. In addition, protegrin-1 (PG-1), another porcine cathelicidin which forms a two-stranded antiparallel beta sheet structure stabilized by two disulfide bonds, has been studied extensively to understand the biological function and acting mechanism of the molecule [24,25]. Although their structures and potency of bactericidal activity are different, they all showed broad spectrum antimicrobial and membranolytic activities. Nisin, a bacteriocin from *Lactococcus lactis*, has also been extensively studied as an effective natural AMP in food industry [26]. Considering the characteristics of the membranolytic activities of the above AMPs, we evaluated their applicability as universal membranolytic agents for the total RNA and genomic DNA isolation of various bacterial strains by comparing the results with those of currently available membranolytic enzymes [27]. In this study, we showed that PMAP-36 could be used as a cell lysis-inducing agent for microbiological studies, especially for hard-to-lyse bacteria, and demonstrated a broader spectrum of lytic activity against various bacterial species than other bacterial cell wall lytic enzymes.

## 2. Results

### 2.1. Successful Production of Recombinant PMAP-36 and PG-1

The two pig cathelicidins, PMAP-36 and PG-1, were selected to evaluate its potential as broad-spectrum bacterial lysis peptides and produced using a previously described AMP expression system [24,28]. The result of protein extraction showed clear expression of the green fluorescent protein (GFP)-PMAP-36 fusion protein with a size of 33 kDa using 12% sodium dodecyl sulfate-polyacrylamide gel electrophoresis (SDS-PAGE) (Figure S1a). The total amount of fusion proteins from a liter of flask culture was approximately 1.3 g for each AMP. Subsequent purification using nickel affinity chromatography (Figure S1b), cyanogen bromide (CNBr) cleavage of target peptides, and final purification using reverse phase-high performance chromatography (RP-HPLC) resulted in the production of 11 to 12 mg of recombinant PMAP-36 and PG-1 with >95% purity (Table 1 and Figure S1c).

### 2.2. Antimicrobial Activity of Cell Wall Lysis-Inducing Proteins

Antimicrobial potency and effective spectrum of cell wall lytic proteins, such as lysozyme, bacteriocins and AMPs, may not be closely related to their bacteriolytic capacity. However, this information may help understand their mechanisms of action and effectiveness as membranolytic agents. We evaluated the bactericidal activities of the commonly used lysis inducible reagents together with AMPs against our bacterial panel consisting of nine bacterial species, including three Gram-negative strains, *Escherichia coli*, *Pseudomonas aeruginosa*, and *Salmonella typhimurium*, and six Gram-positive strains, *Staphylococcus aureus*, *Bacillus cereus*, *Enterococcus faecalis*, *Streptococcus agalactiae*, *Streptococcus dysgalactiae*, and *Streptococcus equi* subsp. *zooepidemicus*.

The analysis showed that PMAP-36 has broad-spectrum antibacterial activities with minimal inhibitory concentration (MIC) values ranging from 3 to 30 μg/mL or 0.7 to 7.2 μM across all nine bacterial species (Table 2). Nisin showed bactericidal activity against all Gram-positive bacteria in our panel, at concentrations ranging from 2 to 16 μg/mL or 600 nM to 3.6 μM. It has been reported that Gram-negative cells are resistant to nisin due to the LPS composition of the outer layer, which acts as a barrier to the action of nisin on the cytoplasmic membrane [29]. Lysostaphin and *Staphylococcus simulans* metalloendopeptidase showed strong activity with an MIC value of 1 μg/mL or 0.2 μM against *S. aureus*, consistent with the application limit of the enzyme. Interestingly, lysozyme showed almost no antimicrobial activity against all tested bacteria even at a concentration of 640 μg/mL.

### 2.3. Cell Wall Lysis-Inducing Activity of PMAP-36 in RNA Isolation

**Figure 1:**
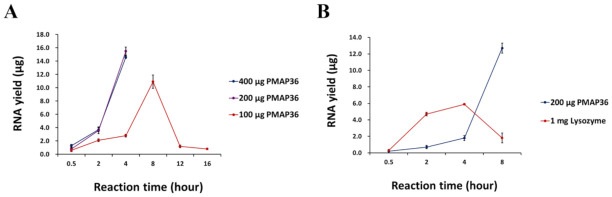
Effects of peptide concentration and reaction time on RNA yields using porcine myeloid antimicrobial peptide 36 (PMAP-36) and lysozyme from *S. aureus* and *S. typhimurium*. (**A**) 1 × 10^9^ cells were added to 150 μL reaction mixture. 100, 200, and 400 μg of PMAP-36 incubated with *S. aureus* for varying reaction times. (**B**) Results from the use of 200 μg PMAP-36 and 1 mg lysozyme for varying reaction times against *S. typhimurium. S. aureus*, *Staphylococcus aureus*; *S. typhimurium*, *Salmonella typhimurium*.

Considering that lysostaphin is an effective cell lysis reagent for isolating nucleic acids from *S. aureus*, a hard-to-lyse bacterial strain, PMAP-36 may be used for applications similar to lysostaphin, with the advantage of markedly broader spectrum bacterial strain applicability than lysostaphin. To test the applicability of PMAP-36 for effectively inducing cell wall lysis for nucleic acid isolation, we selected *S. aureus*, which is a commonly studied hard-to-lyse bacterium, and isolated total RNA under varying concentrations of PMAP-36 and incubation time. The amount of used PMAP-36 was 100, 200, and 400 μg in 150 μL reactions for the lysis of 1 × 10^9^ cells, and the incubation time ranged from 0.5 to 16 h, resulting in a yield of 0.6 ± 0.2 to 15.5 ± 0.6 μg total RNA (Figure 1a). The desirable results were achieved by a maximum of 8 h of incubation. The optimum condition for high quality RNA isolation using PMAP-36 for cell lysis was 200 μg peptides with 4 h of incubation.

To compare the efficiency of PMAP-36 as a cell lytic agent with that of other commonly used lysis methods, such as bead beating and lysostaphin treatment, against *S. aureus*, we conducted RNA extraction using different methods and compared the results (Table 3 and Figure S2). The maximum yield (~17 μg from 1 × 10^9^ cells) was obtained after 30 min of incubation of PMAP-36 followed by bead beating or 4 h of incubation with 200 μg PMAP-36 without bead beating, indicating that PMAP-36 can be used as a cell lysing agent for nucleic acid isolation from *S. aureus* (Table 3 and Table S1). In addition, the yield was similar to that of the lysostaphin method. When the bead beating method was used alone without the treatment of either lytic enzymes or PMAP-36, the yield was lower than that obtained with the use of lytic proteins. In addition, treatment with the combination of lysostaphin and PMAP-36 improved the yield only slightly.

We also tested the applicability of nisin, a bacteria-derived AMP or bacteriocin (Table S1). However, nisin did not effectively lyse staphylococcal cells, including other cells, although nisin showed MIC values equivalent to those of PMAP-36 against most Gram-positive strains. Lysozyme treatment was also not effective for *S. aureus* as previously described (Table 2 and Table S1). RNA isolated using both lysostaphin and PMAP-36 treatments showed RNA integrity number (RIN) values >9.0, appropriate 23S/16S rRNA ratios, and an OD_260_/_280_ ratio >2.0, and these parameters are suitable for most molecular biological applications (Table 3).

### 2.4. Variation in Cell Wall Lytic Activity of PMAP-36 against Gram-Positive Bacteria

The existence of rigid cell walls in Gram-positive bacteria often makes the cells refractory to chemical and enzymatic lysis, leading to failure in obtaining the desired yield and quality of nucleic acids. We compared the efficiency of RNA isolation using several commonly used cell lysis inducers, such as lysozyme, mutanolysin, and lysostaphin, in addition to PMAP-36 and nisin from diverse Gram-positive bacterial species, including *Staphylococcus aureus*, *Bacillus cereus*, *Enterococcus faecalis*, and *Streptococcus dysgalactiae* (Table 4).

RNA yields without any treatment of cell lysis proteins were extremely poor for all Gram-positive strains (Table S2). In contrast, both PMAP-36 and lysostaphin effectively lysed *B. cereus* with an average yield of 21.0 ± 1.6 and 19.1 ± 1.7, respectively. However, PMAP-36 and lysostaphin were ineffective against *E. faecalis* and *S. dysgalactiae*, although PMAP-36 showed slightly better lytic activity than lysostaphin. The treatment of mutanolysin and nisin did not improve the results for the two bacterial strains, indicating the complexity of the cell wall structures of Gram-positive bacteria. Interestingly, lysozyme was able to lyse both *B. cereus* and *E. faecalis* despite its ineffectiveness against other Gram-positive species. However, *S. dysgalactiae* did not show lytic susceptibility to any of the enzymes or AMPs tested. Since *Streptococcus* is a large genus with Gram-positive bacteria, we carried out RNA extraction from other bacteria belonging to this genus (*S. agalactiae*, *S. iniae*, and *S. equi* subsp. *zooepidemicus*) using PMAP-36. However, the results were similar to those of *S. dysgalactiae* (Table S3). Interestingly, the RNA yield from *B. cereus* was higher than that from other Gram-positive bacteria, which could be due to its larger genome and cell size than others (Figure S3).

### 2.5. Broad-Spectrum Cell Wall Lytic Activity of PMAP-36 against Gram-Negative Bacteria

Isolation of nucleic acids from Gram-negative bacteria has been successfully achieved by treating cells with ethylenediaminetetraacetic acid (EDTA) and lysozyme to disrupt the cell wall [27,30]. We compared the efficiency of PMAP-36-induced cell wall lysis to that of lysozyme in the isolation of RNA from commonly used Gram-negative bacteria (e.g., *Escherichia coli*, *Pseudomonas aeruginosa*, and *Salmonella typhimurium*; Table 4). It was observed that either vortexing cells in Tris/EDTA buffer or lysozyme treatment was suitable for efficient RNA isolation from *E. coli* (14.3 ± 0.6 μg or 13.8 ± 0.7 μg from 10^9^ cells) and *P. aeruginosa* (21.1 ± 0.6 or 21.0 ± 0.1) but not *S. typhimurium* (0.3 ± 0.0 or 5.9 ± 0.1) (Table 4 and Table S4), suggesting that the cell wall of *S. typhimurium* differs from the other two bacteria. PMAP-36 treatment under the optimized reaction conditions (4 h of incubation time) was also effective for *E. coli* and *P. aeruginosa* but not for *S. typhimurium* (Table 4). However, the extension of PMAP-36 incubation time to 8 h markedly improved the RNA yield from *S. typhimurium* without decreasing the quality of RNA (RIN = 9.3; Figure S2f). In contrast, lysozyme treatment with 8 h incubation did not improve the results (Figure 1b).

### 2.6. Successful Amplification of the 16S Rrna Gene from RNA Samples Obtained Following PMAP-36 Treatment

To evaluate the quality of the RNA isolated using PMAP-36 treatment, reverse transcription PCR of the 16S rRNA gene from *E. coli*, *P. aeruginosa*, *S. aureus*, and *E. faecalis* was conducted (Figure S4a). Efficient amplification of the target sequence was achieved in all tested samples, indicating that RNA obtained using the PMAP-36 method is suitable for most molecular biological applications, including transcriptomics.

### 2.7. Efficient Isolation of Genomic DNA from S. aureus Using PMAP-36 Treatment

In addition to RNA isolation, we carried out genomic DNA isolation from *S. aureus* using the PMAP-36 and lysostaphin methods. Except for the initial lysis step, the rest of the procedure was identical between the two methods. The final DNA yield and quality were similar between the two methods, although the quality of high molecular weight DNA on the gel appeared slightly better following PMAP-36 treatment than lysostaphin treatment (Table S5 and Figure S4b).

### 2.8. Determination of Minimal Inhibitory Concentration for Melittin and PG-1 and the Estimation of Their Nucletic Acid Isolation Efficiency 

To further evaluate the functionality of AMPs as novel membranolytic agents, we conducted an antimicrobial activity assay and RNA isolation using other well-known membranolytic AMPs, PG-1 and melittin, against our bacterial panel. Melittin showed broad-spectrum antibacterial activities with MIC values ranging from 2 to 45 μg/mL or 0.7 to 15.8 μM across all five bacterial species tested, which is similar to those of PMAP-36. However, the values were much higher for PG-1 (Table S6). When RNA yield was evaluated, the results of the melittin treatment was higher than those of PG-1 except for *S. typhimurium* (Table S7). However, both melittin and PG-1 were not effective on membrane disruption required for RNA isolation against *S. aureus.* These results support that PMAP-36 is most effective in broad spectrum membranolytic activities for the isolation of bacterial nucleic acids among tested AMPs.

### 2.9. Classification of Bacterial Species Depending on Sensitivity to Cell Wall Lytic Proteins

**Figure 2:**
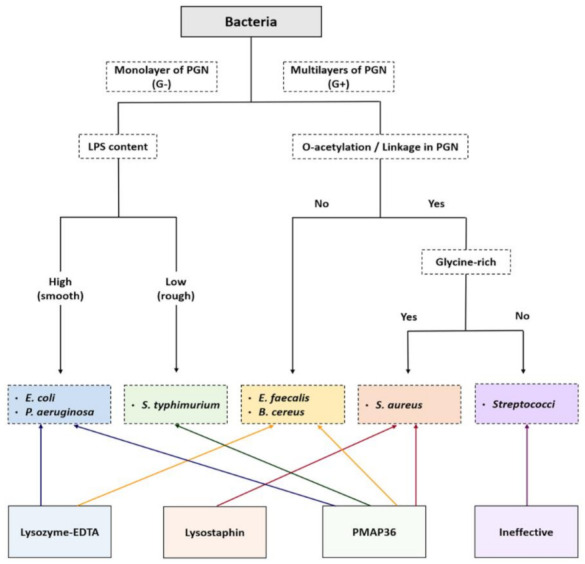
Diagram showing the relationship between the characteristics of bacterial cell walls and susceptibility to membranolytic proteins. Lytic proteins applicable to different bacterial species are shown at the bottom. PGN, peptidoglycan; LPS, lipopolysaccharide; O-acetylation, the N-acetylmuramic acid (NAM) and N-acetyl-D-glucosamine (NAG) linkage by acetylated oxygen; Linkage in PGN, cross-linked wall teichoic acids (WTA); *E. coli*, *Escherichia coli*; *P. aeruginosa*, *Pseudomonas aeruginosa*; *S. typhimurium*, *Salmonella typhimurium*; *E. faecalis*, *Enterococcus faecalis*; *B. cereus*, *Bacillus cereus*; *S. aureus*, *Staphylococcus aureus*.

Differences in the structure and composition of the bacterial outer membrane and cell wall could cause variation in bacterial sensitivity to cell wall lytic proteins. We analyzed similarities and differences among 10 diverse bacterial species for their sensitivity to membranolytic enzymes and AMPs (Table 3, Table 4 and Table S3). The bacterial species employed in this study were classified into five groups depending on the structural characteristics of their cell walls, including proteoglycan layers (monolayer vs. multilayers), lipopolysaccharide (LPS) content (high vs. low), O-acetylation (presence vs. absence), and glycine abundance (rich vs. poor). Next, their experimental sensitivity to cell wall lytic proteins was matched to each group (Figure 2). Interestingly, PMAP-36 showed broad-spectrum sensitivity to the tested bacterial species, except for *E. faecalis* and streptococci.

## 3. Discussion

AMPs have been considered as promising candidates for antibiotic alternatives because of their broad-spectrum antimicrobial activities with less chance of drug resistance and immunomodulatory functions [19]. Some AMPs have also been adopted as cell-penetrating peptides [31]. The bactericidal activity of AMPs is mediated by their ability to form pores on the surface of bacterial cells and disrupt the bacterial membrane [20]. Preparation of high-quality nucleic acids from microorganisms is indispensable for various microbiological studies, including genome analyses and microbiome assays. However, the isolation of genomic DNA or total RNA from hard-to-lyse microorganisms could occasionally be extremely less efficient than easy-to-lyse cells [6,11].

In this study, we tested the possible use of AMPs as bacterial lytic agents for nucleic acid isolation and demonstrated that PMAP-36 could be used as a new bacterial lysis peptide for hard-to-lyse cells independently or in combination with other physical lytic methods. We compared the efficiency of nucleic acid preparation using several commonly used bacterial lysis methods to that of PMAP-36, a potent broad-spectrum AMP, against diverse bacterial species. Our results showed that PMAP-36 or other AMPs with similar properties to PMAP-36 could be effectively used as a new tool for bacterial lysis. Considering the availability of an efficient method to produce recombinant peptides, this method could be more cost effective than using other currently available lytic enzymes for hard-to-lyse bacteria [32]. The availability of cell lysis agents suitable for a wide range of bacterial species, such as PMAP-36, should contribute to decreasing biases from the sample preparation procedure in metagenomics and improving methodological simplicity and convenience.

The responses of bacterial cells to membrane lytic reagents could be an indirect indicator of the structural characteristics of the bacterial cell wall and the outer membrane of a given species. Lysozyme, also known as muramidase or N-acetylmuramide glycanohydrolase, is a glycoside hydrolase that catalyzes the hydrolysis of 1,4-β-linkages between N-acetylmuramic acid (NAM) and N-acetyl-D-glucosamine (NAG) residues in PGN. Lysozyme plus EDTA treatment has been widely used to prepare spheroplasts to destabilize outer membrane LPS structures [33]. Gram-negative bacteria were classified into two groups, “rough” and “smooth,” based on LPS content, which is a determinant of lysozyme-EDTA resistance [3,34]. Smooth bacteria, such as *E. coli* and *P. aeruginosa*, have high LPS content, while *S. typhimurium* has low LPS content and is relatively rough (Figure 2). It has been reported that the carbohydrate part of the LPS component of the outer membrane in *S. typhimurium* is an essential component of a barrier layer that prevents the penetration of large molecules such as antibiotics, lysozyme, and other agents [3]. Among Gram-positive species, *B. cereus* and *E. faecalis* were susceptible to lysozyme even without EDTA (Table 4). However, *S. aureus* and *S. dysgalactiae* showed resistance to lysozyme, which was probably due to the occurrence of O-acetylation and/or linkage with wall teichoic acids at β-D-N-acetylglucosamine, which is the target site of lysozyme [2,35].

The PGN of *S. aureus* consists of a backbone composed of alternating β-1,4 linked NAM and NAG residues, which are cross-linked by tetrapeptide chains consisting of D-alanine, D-glutamine, L-lysine, and D-alanine [36]. These tetrapeptide chains are cross-linked by pentaglycine bridges, which are unique features in the staphylococcal PGN, and confer the cell walls of *S. aureus* with extreme mechanical strength [37]. However, the crosslinking penta-glycine bridges can be cleaved by lysostaphin, which is a glycylglycine endopeptidase found in the PGN of certain staphylococci, including *S. aureus*, *S. simulans*, *S. hyicus*, and *S. xylosus* [37]. Accordingly, the treatment of *S. aureus* with lysostaphin enabled the efficient isolation of nucleic acids from them (Table 3 and Figure S2). However, none of the treatments resulted in efficient cell lysis against *S. dysgalactiae*, although PMAP-36 showed the best results for the strain. The chemical composition of the cell wall of *S. dysgalactiae* may be deficient in glycine or might have modifications and consequently lack the target sites of lysostaphin, in addition to resistance to lysozyme [38,39].

Pretreatment with PMAP-36 prior to performing bead beating resulted in a significant increase in RNA yield from *S. aureus* (Table 3). Therefore, we expect that the use of the same protocol should improve the yield of nucleic acid isolation from *S. dysgalactiae*, which was refractory to both cell wall lytic enzyme- and AMP-induced lysis, although we did not confirm the results. Our results showed that none of the lysis-inducing proteins, including PMAP-36, was universally applicable to all tested bacterial species in this study, although PMAP-36 showed lytic activity against the widest range of bacterial species (Table 4).

Notably, the potency of the antimicrobial activity of PMAP-36 and melittin were not directly related to the efficiency of membrane lysis (Table 2 and Table S6). For example, the MIC value of PMAP-36 was the lowest against *S. dysgalactiae* among bacterial strains in this study, but the efficiency of membrane lysis deduced from RNA yield was poor. In addition, lysozyme showed antimicrobial activity against none of the bacterial species even at extremely high concentrations. Reportedly, lysozyme-induced protoplasts of *Bacillus subtilis* can revert to the bacillary state if they are incubated in a growth medium [40]. 

Vesicle formation on the bacterial cell surface has been detected after PMAP-36 treatment using electron microscopy [22,41]. This phenomenon has also been found in other AMPs with membrane-perturbing activity, such as magainin 2, temporin L, ModoCaths, and SMAP-29 [42,43,44,45]. In addition, the appearance of blebs has been reported as indicative of the ability of temporin L to destabilize the outer membrane of Gram-negative bacteria owing to the displacement of divalent cations that bridge and neutralize LPS [42,46]. This bleb formation on the cell surface has been detected not only in Gram-negative bacteria but also in Gram-positive bacteria [47].

To minimize the amount of PMAP-36 required for bacterial lysis, we determined the optimum incubation time to be 4 h rather than increasing the amount of PMAP-36. The quality of RNA remained intact without degradation even during the long incubation period, of up to 8 h (Figure 1). PMAP-36 activity was also robust to pH changes, which is a preferred characteristic for biological reagents (Table S8).

In this study, we report a novel method for the preparation of large quantities of high-quality nucleic acids from hard-to-lyse bacterial strains using broad-spectrum antimicrobial peptide, PMAP-36. To the best of our knowledge, this is the first study to attempt the exploitation of AMPs for the cell wall lysis of hard-to-lyse bacteria. Our method presents a new option for high quality and unbiased nucleic acid isolation from microbiomes or challenging bacterial strains.

## 4. Materials and Methods

### 4.1. Production of Recombinant PMAP-36 and PG-1

Recombinant PMAP-36 and PG-1 were produced using the GFP-scaffold system described previously [24,28]. Briefly, DL4GFP-AMP-construct transformed BL21 (DE3) cells were grown in 1 L of Luria-Bertani (LB; BD Bioscience, Franklin Lakes, NJ, USA) medium at 37 °C, and the expression of GFP-AMP fusion protein was induced with 0.1 mM isopropyl β-D-1-thiogalactopyranoside at OD_600_ ranging from 0.8 to 1.0. The cells were harvested by centrifugation at 9900× *g* for 10 min at 4 °C after 5 h of induction. The cells were disrupted by sonication (Sonopuls HD 2070; Bandelin, Berlin, Germany) in lysis buffer (20 mM sodium phosphate buffer at pH 7.4 containing 150 mM sodium chloride, 0.1 mM phenylmethylsulfonyl fluoride, and 1 mM dithiothreitol). The insoluble fraction was separated by centrifugation at 20,000× *g* for 20 min at 4 °C. To remove cellular debris, nucleic acids, and cytosolic proteins, DNase (0.01 mg/mL), lysozyme (0.1 mg/mL), and 0.5% Triton-X 100 were added to the insoluble fractions and incubated at room temperature (24 °C) for 20 min. The pellet washes were repeated thrice without the addition of DNase and lysozyme. The insoluble fraction was dissolved in urea buffer (20 mM sodium phosphate buffer at pH 7.4 containing 8 M urea, 500 mM sodium chloride, and 30 mM imidazole), and the target proteins were purified using affinity chromatography using the His Trap HP column (GE Healthcare, Chicago, IL, USA). After dialysis and lyophilization, CNBr was added to the insoluble precipitates in 70% formic acid and incubated to cleave the N- and C-terminal GFPs flanking AMP. After removing CNBr by lyophilization, the target AMP was purified using a preparative RP-HPLC column (DeltaPak C18 Prep column 19, 300 mm; Waters, Tokyo, Japan) in a linear gradient of acetonitrile (5%–90%)/0.1% trifluoroacetic acid for 60 min at a flow rate of 12 mL/min. The target peptides were collected at optical densities of 214 nm and 280 nm. The samples collected before CNBr cleavage and after RP-HPLC were assessed using 12% SDS-PAGE and 16% Tris-Tricine PAGE, respectively, and quantified using the Bradford assay. Purified PG-1 were lyophilized and suspended in 20 mM sodium phosphate buffer pH 7.4 containing 8 M urea, 5 mM reduced glutathione and 0.5 mM oxidized glutathione to install two disulfide bonds within the molecule. The mixture was dialyzed against deionized water and purified peptides were lyophilized. PG-1 and PMAP-36 produced was aliquoted and stored at −20 °C. Chemicals without source information were purchased from Sigma Aldrich (Sigma Aldrich, St. Louis, MO, USA).

### 4.2. Enzymes, Bacterial Strains, and Culture Media

Hen egg white lysozyme, lysostaphin, nisin, mutanolysin, and melittin were purchased from Sigma Aldrich. The following bacteria were used for antimicrobial activity assays and RNA isolation: *Staphylococcus aureus* ATCC 6538 (American Type Culture Collection, Manassas, VA, USA), *Bacillus cereus* ATCC 10876, *Enterococcus faecalis* ATCC 29212, *Streptococcus agalactiae* ATCC 27956, *Streptococcus dysgalactiae* ATCC 27957, *Streptococcus equi* subsp. *zooepidemicus* ATCC 43079, *Streptococcus iniae* KCTC 3657 (Korean Collection for Type Cultures, Daejeon, Korea), *Escherichia coli* ATCC 25922, *Pseudomonas aeruginosa* ATCC 27853, and *Salmonella typhimurium* ATCC 14028. *E. faecalis* and streptococci were cultured in brain heart infusion broth (BD Bioscience) because of their slow growth in LB medium. All other bacteria were cultured in LB medium.

### 4.3. Evaluation of Antibacterial Activity

MIC was determined using a colorimetric method specified by the Microbial Viability Assay Kit-WST (Dojindo, Kumamoto, Japan) according to the manufacturer’s protocol and the Clinical and Laboratory Standards Institute guidelines (2018) [44,48,49,50]. Briefly, four colonies of each bacterium were inoculated into 5 mL LB medium at 37 °C for 4–6 h, allowing the bacteria to reach the log phase. The cells were washed twice with sterile saline (0.9% NaCl) and seeded onto a single well of a 96-well plate at a cell density of 105 CFU/well. Subsequently, 180 μL/well of fresh Mueller Hinton Broth (MHB; BD Bioscience) was added to the plate. Different concentrations of each peptide (protein) and reference antibiotics were serially diluted in 10 μL of MHB and added to each well. The plate was incubated at 37 °C for 6 h. Subsequently, 10 μL of the coloring reagent was added, and the cells were incubated at 37 °C for 2 h. Ampicillin and gentamicin sulfate (Sigma Aldrich) were used as controls for antibacterial activity. UV absorbance was measured in each well at 450 nm using a microplate spectrophotometer (xMark spectrophotometer; Bio-Rad, Hercules, CA, USA). MIC values were determined when the difference in the absorbance values between treatments and blanks (media and coloring reagent only) decreased to <0.05. These experiments were performed in triplicate. The MIC values of PMAP-36 in different pH conditions (pH 5, 6, and 7) adjusted with acetic acid (Sigma Aldrich) were evaluated against *E. coli* (ATCC 25922).

### 4.4. Bacterial Culture and Preparation

A single colony of each strain in a panel of bacteria was cultured in proper medium at 37 °C until they reached mid-log or early stationary phase for RNA or genomic DNA (gDNA) extraction, respectively. The cells were harvested by centrifugation at 3100× *g* and 4 °C for 7 min and washed with sterile ice-cold TE buffer (10 mM Tris-HCl at pH 7.4 and 1 mM EDTA). For *P. aeruginosa*, sterile ice-cold saline (0.9% sodium chloride) was used to wash the pellets to prevent pellet disruption due to their high sensitivity to EDTA. Approximately 1 × 10^9^ cells were used for RNA and genomic DNA isolation.

### 4.5. RNA Isolation Using Different Cell Lysis Methods

#### 4.5.1. Cell Lysis Using Lytic Proteins

After cell preparation, the cells were suspended in 150 μL of RNase-free water or Tris-EDTA buffer (10 mM Tris-HCl at pH 7.4 and 1 mM EDTA) containing cell wall lytic proteins (1 and 5 mg lysozyme, 200 μg lysostaphin, 100 μg nisin, 1 mg mutanolysin, 100–400 μg PMAP-36, and 200 μg melittin and PG-1). The reactions were incubated for 0.5–16 h at 37 °C. Subsequently, 1 mL of TRIzol (Invitrogen, Carlsbad, CA, USA) was added to the cell suspension and incubated at room temperature for 5 min. The reactants were vortexed for 5 min at maximum speed and incubated at room temperature for 5 min. Subsequently, 200 μL chloroform was added to the lysates and mixed gently by inversion. The lysates were centrifuged at 20,000× *g* for 15 min after incubation at room temperature (24 °C) for 5 min. The supernatant (600 μL) was collected and transferred to new tubes. Then, an equal volume of 75% ethanol was added to each tube and inverted several times. The mixtures were transferred to a spin column (RNeasy Mini Kit [Qiagen, Venlo, The Netherlands]), and RNA was extracted according to the manufacturer’s instructions.

#### 4.5.2. Cell Lysis Using Bead Beating

After the cells were prepared, bead beating was performed using a 3 mm Tungsten Carbide Bead (Qiagen) at 25 Hz for 3 min in Tissuelyser II (Qiagen). Subsequently, 1 mL of TRIzol reagent (Invitrogen) was added to the lysates and incubated at 60 °C for 5 min. RNA was extracted in the same manner as described above.

### 4.6. DNA Isolation and Purification

After cell preparation was achieved, the cells were suspended in 150 μL of DNase-free water or Tris-EDTA buffer (10 mM Tris-HCl at pH 7.4 and 1 mM EDTA) containing 200 μg PMAP-36. The reactions involved incubation for 4 h at 37 °C. Subsequently, 200 μL lysis buffer (BL buffer) from GeneAll^®^ ExgeneTM Cell SV mini kit (GeneAll Biotechnology, Seoul, Korea) was added to the cell suspension and vortexed for 5 min at maximum speed. About 20 μL of proteinase K (20 mg/mL) provided by the manufacturer was added to the lysates in the lysis buffer, incubated at 56 °C for 30 min, and then incubated for an additional 30 min at 70 °C. Absolute ethanol (200 μL) was added to the samples, mixed thoroughly, and transferred to the column. Genomic DNA was extracted using the GeneAll^®^ ExgeneTM Cell SV mini kit (GeneAll Biotechnology) according to the manufacturer’s protocol.

### 4.7. Estimation of Nucleic Acid Quality and Quantity

The concentration and purity of the nucleic acids were measured using a NanoDrop^®^ ND-1000 Spectrophotometer (Thermo Scientific, Waltham, MA, USA). Additionally, the RIN values were estimated using a 4200 TapeStation System (Agilent, Santa Clara, CA, USA). DNA integrity was estimated by electrophoresing the isolated DNA on a 1.5% agarose gel in 0.5 × Tris-acetate-EDTA (TAE) buffer. After electrophoresis, the gels were stained with ethidium bromide (Sigma Aldrich) and visualized under UV light.

### 4.8. Amplification of the 16S rRNA Gene

Reverse transcription of purified RNA was conducted using a QuantiTect^®^ Reverse Transcription kit (Qiagen) using 2 µg of total RNA according to the manufacturer’s instructions. A pair of universal primers (5′-TGCCAGCAGCCGCGGTAA-3′ and 5′-CCCGGGAACGTATTCACCGTAGC-3′) to amplify the 16S rRNA gene (GenBank accession no. L37597.1) from all bacterial species in this study was designed using PerlPrimer (v1.1.21) [51]. A 20 μL solution containing 10 pmol of each primer, 1 μL cDNA, 1 μL dNTP (each 2.5 mM), 2 μL 10x reaction buffer (25 mM MgCl2, mixed), and 0.5 U Taq polymerase (Solgent, Daejeon, Korea) was used to perform PCR in Thermocycler 3000 (Biometra, Jena, Germany). The cycling conditions for PCR were as follows: an initial denaturation step at 95 °C for 3 min, followed by 29 cycles of denaturation at 95 °C for 30 s, primer annealing at 67 °C for 45 s, elongation at 72 °C for 45 s, and a final extension at 72 °C for 7 min. The PCR mixture without cDNA was used as a negative control. The results were confirmed by loading 5 μL of the PCR product into 1.5% agarose gel (Sigma Aldrich) containing 0.5 × TAE buffer and electrophoresing at 100 V for 30 min. The gel was stained with ethidium bromide (Sigma Aldrich) and visualized under UV light.

## 5. Conclusions

In this study, we evaluated a novel idea of using broad-spectrum antimicrobial peptides (AMPs) to achieve efficient lysis of hard-to-lyse bacteria for high-quality nucleic acid isolation. We compared the bacteriolytic efficiency of several AMPs including PMAP-36, nisin, melittin, and PG-1 to that of known membranolytic enzymes including lysozyme, mutanolysin, and lysostaphin against ten different bacterial species and presented the results. We discussed the characteristics of different bacterial species on susceptibility to different AMPs and bacteriolytic enzymes. Our results, which employ broad-spectrum AMPs for bacterial lysis, may present a new idea to achieve high quality and unbiased nucleic acid isolation for microbiome studies or for challenging bacterial strains. 

## 6. Patents

The method in this study for nucleic acid isolation using AMPs are the subject of domestic and foreign patent applications by Konkuk University.

## Figures and Tables

**Table 1:** Yield of recombinant PMAP-36 and PG-1 at various steps of purification.

	Yield (mg/L) *	
Purification Step	PMAP-36	PG-1
Total insoluble proteins	1320	1350
Ni-NTA purification	241	239
RP-HPLC	11.4	12.1

* The yields of each purification step were determined using Bradford assay; Ni-NTA, nickel nitrilotriacetic acid. PG-1 = protegrin-1. PMAP-36 = porcine myeloid antimicrobial peptide 36.

**Table 2:** Comparison of antimicrobial activities among lysozyme, lysostaphin, nisin, and PMAP-36 against bacterial strains examined in this study.

	Strain	Minimal Inhibitory Concentration (μg/mL, (μM))					
		PMAP-36	Lysozyme	Lysostaphin	Nisin	Ampicillin ^b^	Gentamicin ^b^
Gram-positive bacteria	*S. aureus* ATCC 6538	6 (1.4)	>640 (44.8) ^a^	1 (0.2)	2 (0.6)	2 (5.7)	1 (2.1)
	*B. cereus* ATCC 10876	22 (5.3)		>160 (29.7) ^a^	4 (1.2)	80 (228.8)	1 (2.1)
	*E. faecalis* ATCC 29212	20 (4.8)			16 (3.6)	10 (28.6)	90 (189.0)
	*S. agalactiae* ATCC 27956	5 (1.2)			2 (0.6)	4 (11.4)	75 (157.5)
	*S. dysgalactiae* ATCC 27957	3 (0.7)			4 (1.2)	2 (5.7)	15 (31.5)
	*S. equi* subsp. *zooepidemicus* ATCC 43079	11 (2.6)			4 (1.2)	2 (5.7)	45 (94.5)
Gram-negative bacteria	*E. coli* ATCC 25922	6 (1.4)	>640 (44.8) ^a^	>160 (29.7) ^a^	>64 (19.2)	5 (14.3)	1 (2.1)
	*P. aeruginosa* ATCC 27853	6 (1.4)				>640 (1831.7)	1 (2.1)
	*S. typhimurium* ATCC 14028	30 (7.2)				42.5 (121.6)	6 (12.6)

^a^ The values apply to all bacterial strains within each section of the column. ^b^ Antibiotics for comparison.

**Table 3:** Comparison of RNA yield from *S. aureus* using different cell wall lysis methods.

	Yield		
Treatment	Amount (μg)	RIN *	23S/16S rRNA Ratio
200 μg PMAP-36 for 4 h	15.5 ± 0.6	9.3	1.9
200 μg Lysostaphin for 30 min	16.9 ± 1.2	9.3	1.6
Bead beating	10.3 ± 1.2	8.3	1.0
200 μg Lysostaphin + 200 μg PMAP-36 for 30 min	17.8 ± 0.1	9.0	1.4
200 μg PMAP-36 for 30 min + bead beating	16.8 ± 1.3	9.3	1.7
No treatment	0.7 ± 0.2	N.D.	N.D.

* The corresponding electropherograms are shown in Figure S2. RIN = RNA integrity number. N.D., Not determined.

**Table 4:** Comparison of RNA extraction results using different cell lysis methods for diverse bacterial species.

			Yield	Optical Density	
Strain		Treatment	Amount (μg)	A_260_/A_280_	A_260_/A_230_
Gram-positive bacteria	*B. cereus* ATCC 10876	No treatment	0.5 ± 0.1	1.39 ± 0.04	1.32 ± 0.09
		5 mg Lysozyme for 30 min	21.5 ± 1.2	2.06 ± 0.01	2.05 ± 0.10
		200 μg Lysostaphin for 30 min	19.1 ± 1.7	2.10 ± 0.04	2.22 ± 0.14
		200 μg PMAP-36 for 4 h	21.0 ± 1.6	2.14 ± 0.02	2.13 ± 0.03
	*E. faecalis* ATCC 29212	No treatment	0.8 ± 0.1	1.38 ± 0.05	0.46 ± 0.34
		5 mg Lysozyme for 30 min	11.3 ± 0.5	2.11 ± 0.02	2.18 ± 0.07
		200 μg Lysostaphin for 30 min	0.7 ± 0.2	1.36 ± 0.04	0.57 ± 0.43
		200 μg PMAP-36 for 4 h	3.2 ± 0.3	1.76 ± 0.05	1.45 ± 0.08
	*S. dysgalactiae* ATCC 27957	No treatment	0.5 ± 0.3	1.40 ± 0.09	0.57 ± 0.31
		5 mg Lysozyme for 30 min	0.4 ± 0.1	1.27 ± 0.05	0.96 ± 0.39
		200 μg Lysostaphin for 30 min	0.4 ± 0.1	1.34 ± 0.07	0.52 ± 0.29
		200 μg PMAP-36 for 4 h ^a^	2.3 ± 0.5	1.54 ± 0.09	0.66 ± 0.16
Gram-negative bacteria	*E. coli* ATCC 25922	No treatment	14.3 ± 0.6	2.17 ± 0.01	2.23 ± 0.01
		1 mg Lysozyme for 30 min	13.8 ± 0.7	2.13 ± 0.03	2.04 ± 0.21
		200 μg PMAP-36 for 4 h	14.6 ± 0.6	2.16 ± 0.04	2.05 ± 0.15
	*P. aeruginosa* ATCC 27853 ^b^	No treatment	21.1 ± 0.6	1.83 ± 0.06	1.29 ± 0.54
		1 mg Lysozyme for 30 min	21.0 ± 0.1	2.11 ± 0.02	2.24 ± 0.02
		200 μg PMAP-36 for 4 h	22.3 ± 0.6	2.2 ± 0.01	2.04 ± 0.11
	*S. typhimurium* ATCC 14028	No treatment	1.5 ± 0.2	1.67 ± 0.01	0.67 ± 0.48
		1 mg Lysozyme for 4 h	5.9 ± 0.1	2.07 ± 0.03	1.97 ± 0.09
		200 μg PMAP-36 for 8 h	12.7 ± 0.6	2.07 ± 0.05	2.09 ± 0.03

^a^ RNA yields were improved when RNase free water was used as a reaction buffer (Tables S2 and S3). ^b^ RNA yields were improved when Tris-EDTA buffer was used as a reaction buffer (Tables S2 and S3).

## Data Availability

The datasets used in this study can be found in the GenBank database under the accession numbers: CP009072.1, CP011857.1, AL513382.1, NZ_CP020020.1, CM000715.1, CP008816.1, CM001076.1. The data supporting the conclusions of current article will be available in Supplementary Materials by the authors.

## Supplementary Materials

The following are available online at https://www.mdpi.com/article/10.3390/ijms22084149/s1, Figure S1: AMP production, Figure S2: Electropherogram images from the TapeStation System (Agilent) to estimate RNA quality using different cell lysis methods, Figure S3: Results showing the relationship between total RNA yields and bacterial genome sizes, Figure S4: Gel images showing the results of electrophoretic analysis on extracted nucleic acids, Table S1: Additional data on RNA isolation using lysozyme and nisin from *S. aureus* ATCC 6538, Table S2: Additional data for the optimization of bacterial lysis methods for RNA isolation against different bacterial species, Table S3: Analysis of the effect of reaction buffers for RNA extraction from streptococci using PMAP-36, Table S4: Comparison of RNA isolation efficiency using lysozyme in different conditions from *S. typhimurium*, Table S5: Comparison of the efficiency of genomic DNA isolation from *S. aureus* between lysostaphin and PMAP-36 methods, Table S6: Antimicrobial activities of PG-1 and melittin to hard-to-lyse bacterial strains, Table S7: The results of RNA extraction using PG-1and melittin from hard-to-lyse bacteria, Table S8: Antimicrobial activity of PMAP-36 against *E. coli* ATCC 25922 under different pH conditions.
