# Supplementary material for: High-Quality Nucleic Acid Isolation from Hard-to-Lyse Bacterial Strains Using PMAP-36, a Broad-Spectrum Antimicrobial Peptide
Source: Int J Mol Sci. 2021 Apr 16;22(8):4149. doi: 10.3390/ijms22084149 (PMC8073543; doi:10.3390/ijms22084149)
Supplement: Supplementary file 1 [file ijms-22-04149-s001.zip › ijms-1186445-supplementary.pdf]

International Journal of
Molecular Sciences

MDPI

Article

High-Quality Nucleic Acid Isolation from Hard-to-Lyse Bacterial Strains Using PMAP-36, a Broad-Spectrum Antimicrobial Peptide

Hye-sun Cho, Munjeong Choi, Yunjung Lee, Hyoim Jeon, Byeongyong Ahn, Nagasundarapandian Soundrarajan, Kwonho Hong, Jin-Hoi Kim and Chankyu Park

Supplementary information

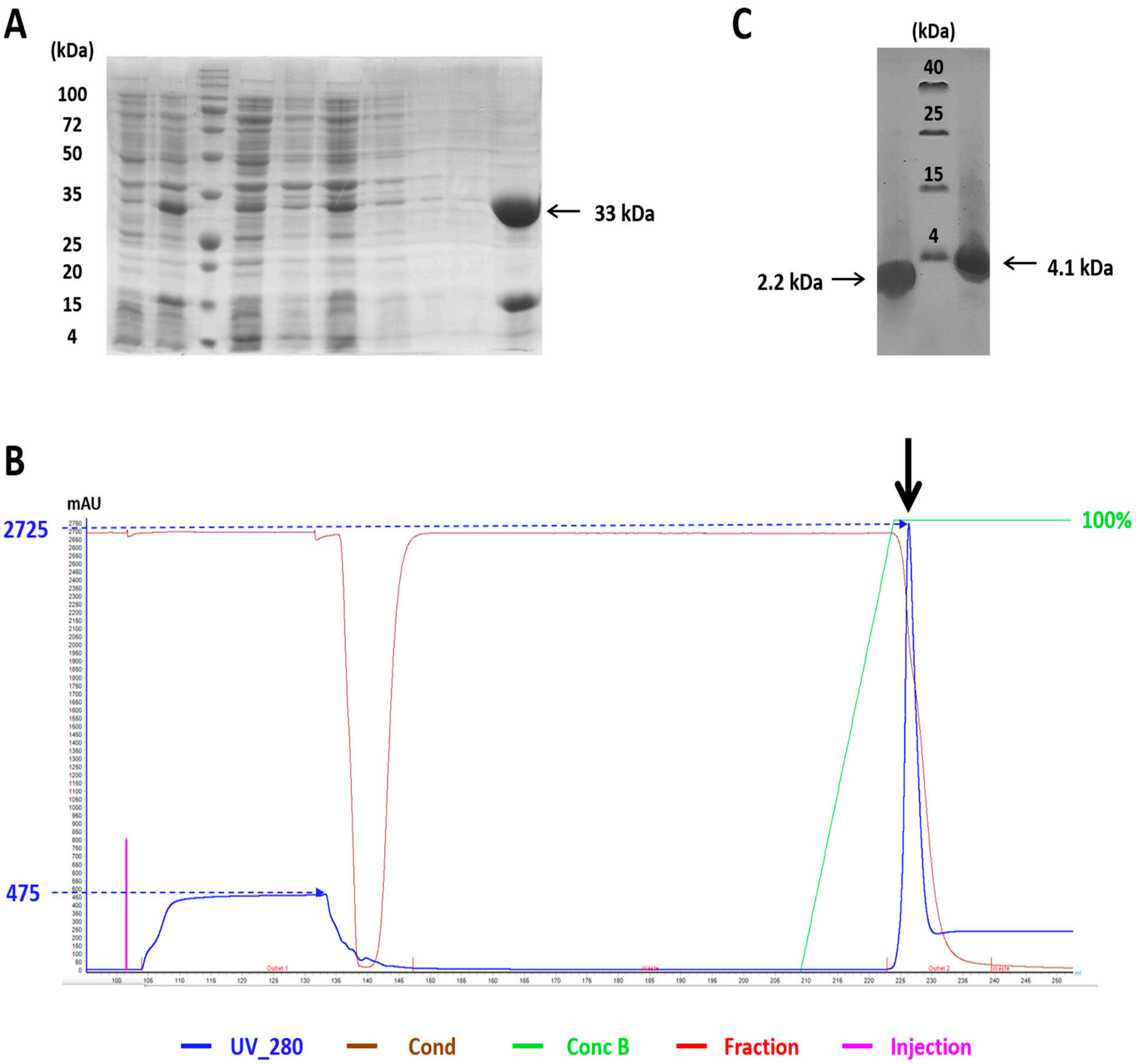

Figure S1. AMP production. **(A)** A polyacrylamide (12% SDS-PAGE) gel image showing DL4GFP-PMAP-36 insoluble extracts (33 kDa) indicated by an arrow. **(B)** The results of Ni affinity chromatography showing the target peak indicated by an arrow. **(C)** The images of purified PG-1 (2.2 kDa) and PMAP-36 (4.1 kDa) separated in 16% Tris-Tricine PAGE and stained with Coomassie blue.

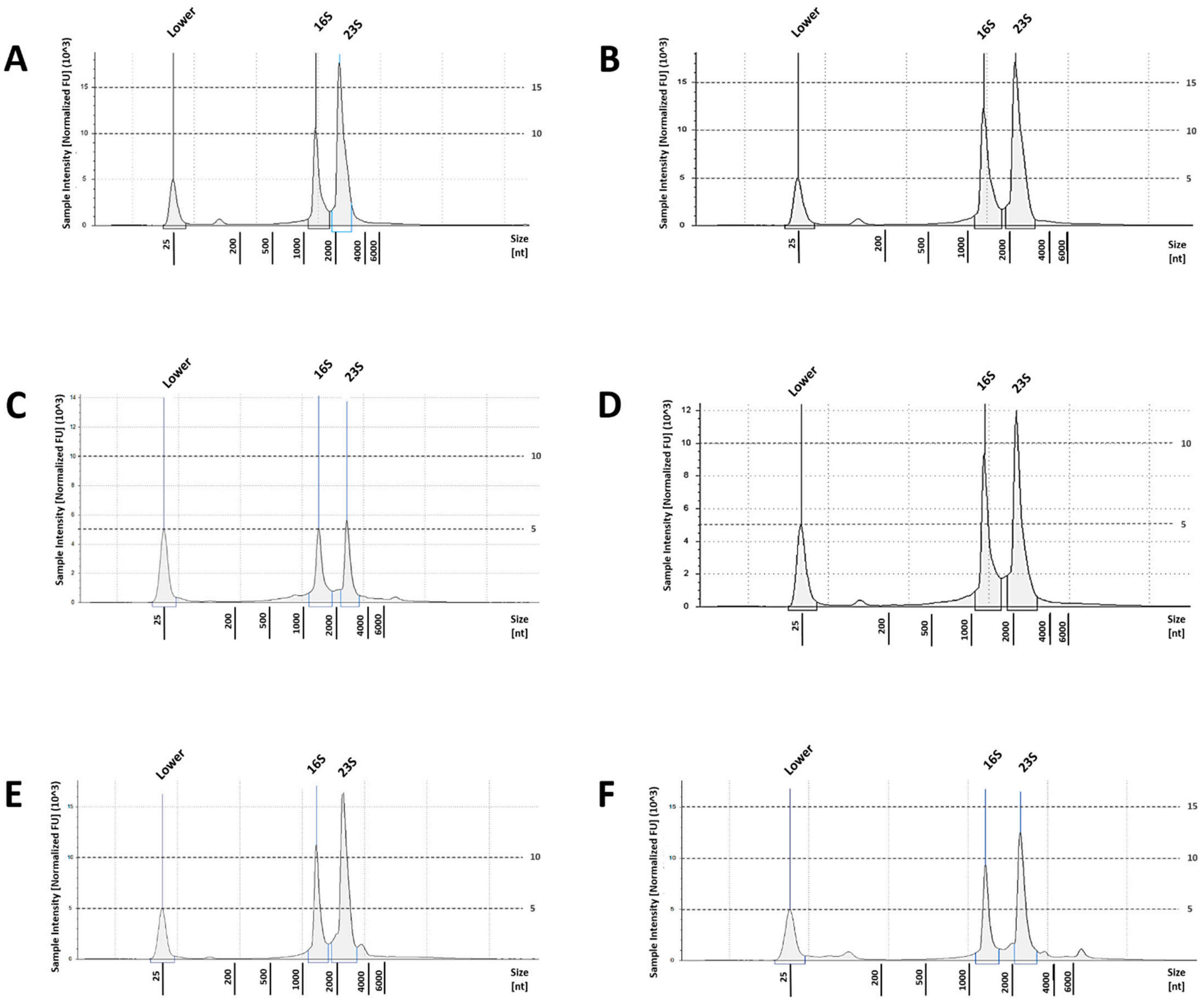

Figure S2. Electropherogram images from the TapeStation System (Agilent) to estimate RNA quality using different cell lysis methods. RNA was isolated from *S. aureus* ATCC 6538 (1×10^9 cells). The results from 200 µg PMAP-36 for 4 h **(A)**, 200 µg Lysostaphin for 30 min **(B)**, bead beating only **(C)**, 200 µg Lysostaphin + 200 µg PMAP-36 for 30 min **(D)**, and 200 µg PMAP-36 peptide treatment for 30 min combined with subsequent bead beating **(E)** methods were compared. The RNA quality from *S. typhimurium* ATCC 14028 using 200 µg PMAP-36 for an 8 h reaction is shown in **(F)**. RIN values for A to F were 9.3, 9.3, 8.3, 9.0, 9.3, and 9.3, respectively. *S. aureus*, *Staphylococcus aureus*; *S. typhimurium*, *Salmonella typhimurium.*

A

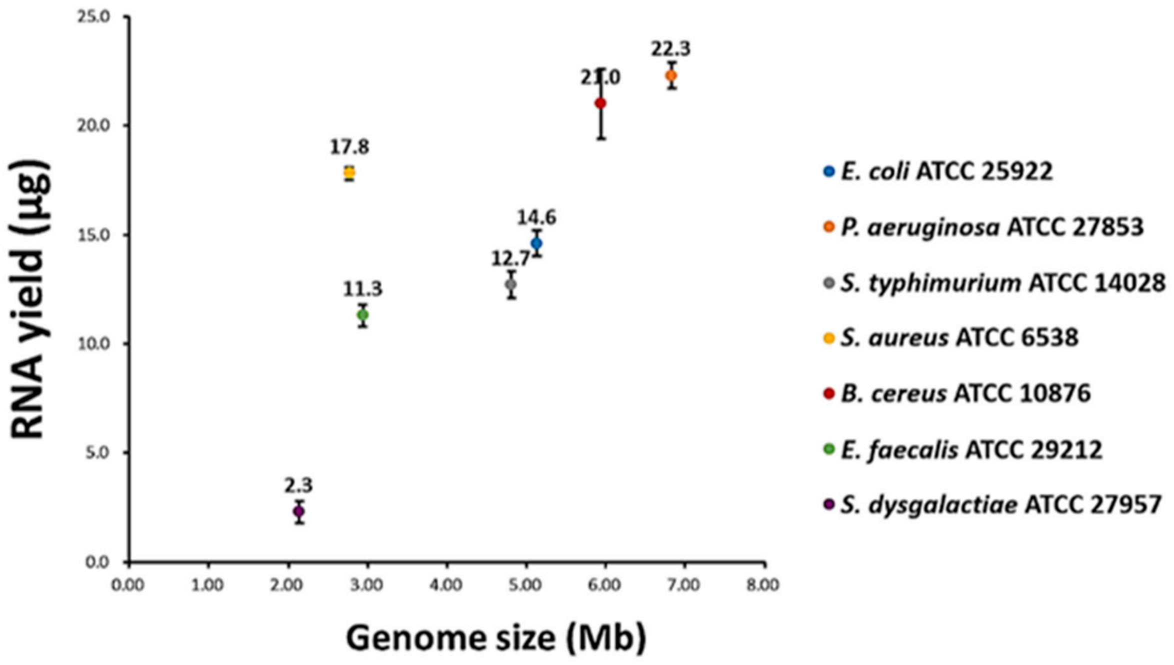

B

Strain	Genome size (Mb)	GenBank accession number	Cell size (µm)	Reference
E. coli ATCC 25922	5.13	CP009072.1	1 to 2 x 0.5	National Academy of Sciences, 1999
P. aeruginosa ATCC 27853	6.83	CP011857.1	1 to 5 x 0.5 to 1.0	Lederberg et al., 2000
S. typhimurium ATCC 14028	4.81	AL513382.1	2 to 5 x 0.5 to 1.5	Public Health Agency of Canada, 2010
S. aureus ATCC 6538	2.77	NZ_CP020020.1	1	Monteiro et al., 2015
B. cereus ATCC 10876	5.94	CM000715.1	3 to 5 x 1	Stecchini et al., 2009
E. faecalis ATCC 29212	2.94	CP008816.1	0.6 to 2.0	Oyama et al., 2017
S. dysgalactiae ATCC 27957	2.14	CM001076.1	1.07 to 1.21	Kokkinosa et al., 1998

Figure S3. Results showing the relationship between total RNA yields and bacterial genome sizes. **(A)** The bacTable 2. 76). **(B)** Cell and genome sizes of the bacteria used are shown. The cell sizes for rod and spherical bacteria are indicated by "length × width" and "diameter," respectively. *E. coli*, *Escherichia coli*; *P. aeruginosa*, *Pseudomonas aeruginosa*; *S. typhimurium*, *Salmonella typhimurium*; *E. faecalis*, *Enterococcus faecalis*; *B. cereus*, *Bacillus cereus*; *S. aureus*, *Staphylococcus aureus*; *S. dysgalactiae*, *Streptococcus dysgalactiae*.

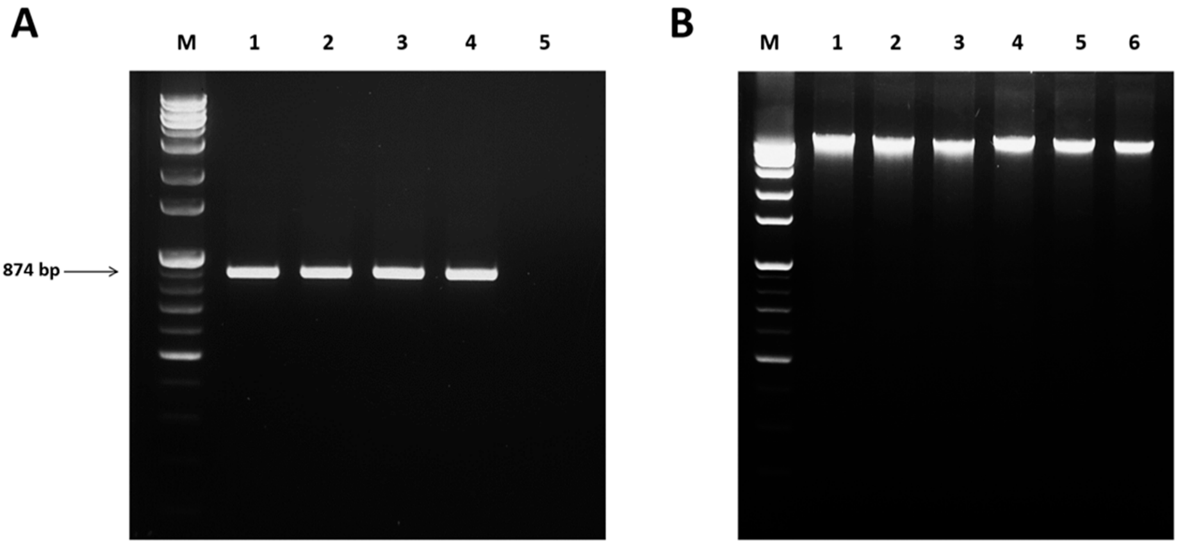

Figure S4. Gel images showing the results of electrophoretic analysis on extracted nucleic acids. **(A)** Results of the analysis of 16S rRNA amplicons from the reverse transcription PCR of RNA isolated using PMAP-36 from *E. coli*, *P. aeruginosa*, *S. aureus*, *E. faecalis*, and a negative control (lanes 1 to 5, respectively). The amplicons are indicated by an arrow on the left. **(B)** The electrophoretic image of genomic DNA isolated using lysostaphin (lane 1 to 3) and PMAP-36 (lane 4 to 6) treatments from *S. aureus*. A total of 200 ng of DNA was loaded. *E. coli*, *Escherichia coli*; *P. aeruginosa*, *Pseudomonas aeruginosa*; *S. aureus*, *Staphylococcus aureus*; *E. faecalis*, *Enterococcus faecalis*.

Table S1. Additional data on RNA isolation using lysozyme and nisin from *S. aureus* ATCC 6538.

Treatment	Yield	Optical density	
	Amount (μg)	A_{260}/A_{280}	A_{260}/A_{230}
5 mg Lysozyme for 30 min	0.1±0.1	1.60±0.01	0.40±0.15
100 μg Nisin for 30 min	0.9±0.3	1.45±0.05	1.03±0.11
100 μg Nisin for 4 h	2.7±0.3	1.68±0.05	1.23±0.07
5 mg Lysozyme + 200 μg PMAP-36	1.2±0.1	1.71±0.03	0.56±0.10

Table S2. Additional data for the optimization of bacterial lysis methods for RNA isolation against different bacterial species.

Strain	Treatment [a]	Reaction buffer [b]	Yield	Optical density	
			Amount (μg)	A_{260}/A_{280}	A_{260}/A_{230}
B. cereus ATCC 10876	No treatment	TE or W	0.5±0.1	1.39±0.04	1.32±0.09
E. faecalis ATCC 29212	No treatment	TE or W	0.8±0.1	1.38±0.05	0.46±0.34
S. dysgalactiae ATCC 27957	No treatment	TE or W	0.5±0.3	1.40±0.09	0.57±0.31
	5 mg Mutanolysin for 30 min	TE or W	0.5±0.1	1.38±0.07	0.98±0.02
	200 μg Nisin for 4 h	TE or W	0.6±0.1	1.11±0.03	0.87±0.32
	200 μg PMAP-36 for 4 h	W	2.3±0.5	1.54±0.09	0.66±0.16
		TE	0.9±0.3	1.48±0.01	0.55±0.14
P. aeruginosa ATCC 27853	No treatment	W	0.7±0.2	1.23±0.02	0.57±0.21
		TE	21.1±0.6	1.83±0.06	1.29±0.54
	1 mg Lysozyme for 30 min	W	1.2±0.1	1.43±0.03	1.42±0.23
		TE	21.0±0.1	2.11±0.02	2.24±0.02
	200 μg PMAP-36 for 4 h	W	2.1±0.1	1.29±0.04	1.04±0.13
		TE	22.3±0.6	2.2±0.01	2.04±0.11
S. typhimurium ATCC 14028	No treatment	TE or W	1.5±0.2	1.67±0.01	0.67±0.48

[a] A 5 min vortexing was carried out after all treatments. No treatment corresponds to vortexing only without any treatment.
[b] Tris-EDTA buffer and RNase-free water are indicated as "TE" and "W," respectively.

Table S3. Analysis of the effect of reaction buffers for RNA extraction from streptococci using PMAP-36.

Strain	Reaction buffer #	Yield	Optical density	
		Amount (µg)	A_{260}/A_{280}	A_{260}/A_{230}
S. agalactiae ATCC 27956	TE	0.8±0.2	1.23±0.08	0.79±0.03
	W	2.0±0.3	1.67±0.01	0.59±0.23
S. dysgalactiae ATCC 27957	TE	0.9±0.3	1.48±0.01	0.55±0.14
	W	2.3±0.5	1.54±0.09	0.66±0.16
S. iniae KCTC 3657	TE	0.5±0.1	1.28±0.02	0.51±0.07
	W	1.6±0.4	1.61±0.06	0.46±0.25
S. equi subsp. zooepidemicus ATCC 43079	TE	0.6±0.2	1.23±0.08	0.79±0.03
	W	1.7±0.1	1.70±0.06	0.88±0.05

The RNA isolation efficiency of 200 µg of PMAP-36 was tested for 4 h of incubation time at 37 °C. Tris-EDTA buffer and RNase-free water are indicated as "TE" and "W," respectively.

Table S4. Comparison of RNA isolation efficiency using lysozyme in different conditions from *S. typhimurium.*

Amount of lysozyme (mg)	Reaction time (h)	Concentration of EDTA (mM)	Yield	Optical density	
			Amount (µg)	A_{260}/A_{280}	A_{260}/A_{230}
	0.5		1.2±0.2	1.38±0.05	1.16±0.35
**5**	2.0	1	3.4±0.9	2.02±0.03	1.80±0.06
	4.0		4.5±0.0	2.08±0.02	1.63±0.64
	0.5		0.3±0.0	1.97±0.11	1.20±0.78
1	2.0	1	4.7±0.2	2.09±0.01	1.43±0.92
	4.0		5.9±0.1	2.07±0.03	1.97±0.09
	0.5		3.0±0.1	1.96±0.10	0.78±0.33
1	2.0	5	3.0±0.3	2.01±0.08	1.11±0.66
	4.0		3.2±0.2	2.07±0.02	1.29±0.31
	0.5		1.5±0.1	1.96±0.12	1.12±0.27
1	2.0	10	1.5±0.0	2.05±0.02	1.44±0.40
	4.0		1.9±0.4	1.97±0.11	0.82±0.61

Table S5. Comparison of the efficiency of genomic DNA isolation from *S. aureus* between lysostaphin and PMAP-36 methods.

Treatment		Yield	Optical density	
Lytic protein	Vortexing	Amount (µg)	A_{260}/A_{280}	A_{260}/A_{230}
200 µg Lysostaphin for 30 min	None	16.9±1.8	2.05±0.03	2.20±0.25
200 µg PMAP-36 for 4 h	None	5.5±0.8	1.34±0.01	0.60±0.01
200 µg PMAP-36 for 4 h	5 min	17.0±2.1	2.11±0.03	2.13±0.21

Table S6. Antimicrobial activities of PG-1 and melittin to hard-to-lyse bacterial strains.

Strains		MIC (µg/mL, µM)	
		PG-1	Melittin
Gram-positive bacteria	*S. aureus* ATCC 6538	43 (19.9)	2 (0.7)
	B. cereus ATCC 10876	> 160 (74.1)	10 (3.5)
	E. faecalis ATCC 29212	160 (74.1)	3.5 (1.2)
	S. dysgalactiae ATCC 27957	31.5 (14.6)	7 (2.58)
Gram-negative bacteria	*S. typhimurium* ATCC 14028	85 (39.4)	45 (15.8)

Table S7. The results of RNA extraction using PG-1 and melittin from hard-to-lyse bacteria.

Strain		Treatment	Yield	Optical density	
			Amount (μg)	A_{260}/A_{280}	A_{260}/A_{230}
Gram-positive bacteria	*S. aureus* ATCC 6538	200 μg PG-1 for 4 h	1.6±0.2	1.88±0.09	1.24±0.06
		200 μg Melittin for 4 h	2.2±0.1	1.85±0.11	1.85±0.03
	B. cereus ATCC 10876	200 μg PG-1 for 4 h	8.7±0.1	2.10±0.01	2.03±0.15
		200 μg Melittin for 4 h	21.2±0.1	2.16±0.01	2.17±0.09
	E. faecalis ATCC 29212	200 μg PG-1 for 4 h	3.0±0.4	1.87±0.06	1.54±0.06
		200 μg Melittin for 4 h	3.3±0.2	1.97±0.06	1.71±0.10
	S. dysgalactiae ATCC 27957	200 μg PG-1 for 4 h	0.9±0.1	1.51±0.01	1.46±0.19
		200 μg Melittin for 4 h	1.2±0.2	1.52±0.07	1.09±0.14
Gram-negative bacteria	*S. typhimurium* ATCC 14028	200 μg PG-1 for 4 h	8.1±0.4	2.09±0.02	1.96±0.04
		200 μg PG-1 for 8 h	7.4±0.1	2.12±0.01	2.16±0.07
		200 μg Melittin for 4 h	7.4±0.1	2.05±0.03	1.97±0.16
		200 μg Melittin for 8 h	4.7±0.6	2.04±0.01	1.96±0.14

Table S8. Antimicrobial activity of PMAP-36 against *E. coli* ATCC 25922 under different pH conditions.

pH	MIC (μg/mL, (μM))
	PMAP-36
5.0	3.5 (1.0)
6.0	4.5 (1.1)
7.0	6.0 (1.4)
7.5	6.0 (1.4)
